# MiR-133b is frequently decreased in gastric cancer and its overexpression reduces the metastatic potential of gastric cancer cells

**DOI:** 10.1186/1471-2407-14-34

**Published:** 2014-01-21

**Authors:** Yu Zhao, Jie Huang, Li Zhang, Ying Qu, Jianfang Li, Beiqin Yu, Min Yan, Yingyan Yu, Bingya Liu, Zhenggang Zhu

**Affiliations:** 1 Shanghai Key Laboratory of Gastric Neoplasms, Department of Surgery, Shanghai Institute of Digestive Surgery, Ruijin Hospital, Shanghai Jiao Tong University School of Medicine, 197 Rui Jin Road, Shanghai 200025, People’s Republic of China

**Keywords:** MicroRNA, miR-133b, Gastric cancer, Metastasis

## Abstract

**Background:**

Emerging evidence has shown that microRNAs are involved in gastric cancer development and progression. Here we examine the role of miR-133b in gastric cancer.

**Methods:**

Quantitative real-time PCR analysis was performed in 140 patient gastric cancer tissues and 8 gastric cancer cell lines. The effects of miR-133b in gastric cancer cells metastasis were examined by scratch assay, transwell migration and matrigel invasion. *In vivo* effects of miR-133b were examined in an intraperitoneal mouse tumor model. Targets of miR-133b were predicted by bioinformatics tools and validated by luciferase reporter analyses, western blot, and quantitative real-time PCR.

**Results:**

MiR-133b was significantly downregulated in 70% (98/140) of gastric cancer patients. Expression of miR-133b was negatively correlated with lymph node metastasis of gastric cancer in patients. Similarly, the expression of miR-133b was significantly lower in seven tested gastric cancer cell lines than in the immortalized non-cancerous GES-1 gastric epithelial cells. Overexpression of miR-133b markedly inhibited metastasis of gastric cancer cells *in vitro* and *in vivo*. Moreover, the transcriptional factor Gli1 was identified as a direct target for miR-133b. Level of Gli1 protein but not mRNA was decreased by miR-133b. Activity of luciferase with Gli1 3′-untranslated region was markedly decreased by miR-133b in gastric cancer cells. Gli1 target genes, OPN and Zeb2, were also inhibited by miR133b.

**Conclusions:**

MiR-133b is frequently decreased in gastric cancer. Overexpression of miR-133b inhibits cell metastasis *in vitro* and *in vivo* partly by directly suppressing expression of Gli1 protein. These results suggested that miR-133b plays an important role in gastric cancer metastasis.

## Background

Based on the GLOBOCAN 2008 estimates, a total of 989,600 new gastric cancer (GC) cases and 738,000 deaths occurred in 2008, accounting for 8% of the total cancer cases and 10% of total cancer deaths worldwide [1]. Despite advances in treatment, the survival rate of patients with GC remains low. There is still a vital need for the development of novel diagnostic and therapeutic strategies for this disease.

MicroRNAs (miRNAs) are a class of small (18–25 nucleotides), endogenous, non-coding RNAs that silence protein expression by interacting with the 3′-untranslated regions (3′UTRs) of target mRNAs. Growing evidence has shown that miRNAs can function as either oncogenes or tumor suppressors in various cancers [2,3]. Several studies have demonstrated that miRNAs play an important role in GC [4,5]. In our previous work, we identified numerous putative miRNAs with different expression levels in GC and normal tissues by comparing the miRNA expression profile of 28 patient samples of GC tissues and adjacent non-tumor tissues [6]. We have confirmed and investigated several miRNAs disregulated in GC, such as miR-126 [7], miR-409-3p [8], miR-625 [9], miR-21 [10], miR-301a [11] and miR-155 [12]. Although many miRNAs have been identified in association with GC, the mechanism of miRNAs in gastric tumorigenesis still needs to be investigated. MiR-133b was one of the most significantly downregulated miRNAs in GC; however it has been rarely investigated in GC. These results were consistent with another group’s finding from miRNA microarray data in three GC patient tissues [13]. MiR-133b was originally suggested as being solely expressed in skeletal muscle [14]. Recently, miR-133b was implicated to function as a tumor suppressor and its levels were decreased in many types of cancers such as head and neck/oral, bladder, non-small cell lung, cervical, colorectal and esophageal squamous cell cancer [15-22].

In this study, we found that the expression of miR-133b was downregulated in 70% (98/140) of the GC tissues, and this downregulation was associated with lymphatic metastasis of GC. We also present the first data demonstrating that miR-133b overexpression could repress the metastasis of GC cells *in vitro* and *in vivo* by directly targeting the Gli1 transcription factor and inhibiting expression of the Gli1 target genes OPN and Zeb2.

## Methods

### Ethics statement

Written informed consent was obtained from all participants. The study was approved by the Human Research Ethics Committee of Ruijin Hospital, School of Medicine, Shanghai Jiao Tong University (HREC 08–028), and the Laboratory Animal Ethics Committee of Ruijin Hospital. Research in human GC tissues was conducted in accordance with the Declaration of Helsinki. Animal procedures were carried out according to the Animal Research: Reporting *In Vivo* Experiments (ARRIVE) guidelines.

### Cell lines and cell culture

Human GC cell lines SGC-7901, NCI-N87, BGC-823, and AGS were purchased from Shanghai Institutes for Biological Sciences, Chinese Academy of Sciences (Shanghai, China). MKN-45 and MKN-28 were obtained from the Japanese Cancer Research Resources Bank (Tokyo, Japan), and KATO III and SNU-1 were originally purchased from the American Type Culture Collection (Manassas, VA, USA). GES-1, an immortalized gastric epithelial cell line, was a gift from Professor Feng Bi (Huaxi Hospital, Sichuan University, Chengdu, China). Cells were stored, recovered from cryopreservation in liquid nitrogen and used at early passages. All cells were maintained in RPMI-1640 medium plus 10% fetal bovine serum (FBS) and cultured in a 5% CO_2_ humidified atmosphere.

### Patient tissues

GC patient tissues and the adjacent non-tumor tissues were obtained from 140 GC patients undergoing radical gastrectomy at the Department of Surgery, Ruijin Hospital, School of Medicine, Shanghai Jiao Tong University. All patients provided consent and samples were confirmed by independent pathological examination. None of the patients received preoperative treatment. The pathologic tumor staging was determined according to the International Union Against Cancer (2009).

### RNA isolation and quantitative real-time PCR (qRT-PCR)

Total RNA was isolated with Trizol reagent (Invitrogen, Carlsbad, CA, USA) following the manufacturer’s instructions. After the quantitation of mRNA, 2 μg of total RNA were reverse transcribed with random primers following the manufacturer’s instructions (MBI Fermentas, Vilnius, Lithuania). The PCR amplifications were performed in triplicate using the SYBR Green Real Time PCR (Applied Biosystems, Foster City, CA, USA) following the manufacturer’s instructions. Quantification was performed using the ΔΔCt relative quantification method with human GAPDH as an internal control. The following primers were used: Gli1 [GenBank:NM_005269.2, GI: 224809486] (sense: 5′-GGA AGT CAT ACT CAC GCC TCG A-3′; antisense: 5′-CAT TGC TGA AGG CTT TAC TGC A-3′) [23], Zeb2 [GenBank: NM_001171653.1, GI: 224809486] (sense: 5′-AGC CAC GAT CCA GAC CGC AA-3′; antisense: 5′- GCT GTG TCA CTG CGC TGA AGG T-3′), OPN [Genbank: NM_000582, GI:38146097] (sense: 5′-GGA TCC CTC ACT ACC ATG AG-3′; antisense: 5′-AAG CTT GAC CTC AGA AGA TGC ACT-3′) [24] and GAPDH [GenBank:NM_002046.4, GI: 284413745] (sense: 5′-GGA CCT GAC CTG CCG TCT AG-3′; antisense: 5′-GTA GCC CAG GAT GCC CTT GA-3′).

The expression levels of miRNAs were assessed by the stem-loop RT-PCR method using the Hairpin-it™ miRNAs qPCR Quantitation Kit (GenePharma, Shanghai, China) with specific primers for miR-133b and U6 small nuclear RNA (RNU6B). Relative miRNA expression of miR-133b was normalized against the endogenous control, U6, using the ΔΔCt method.

### Transient transfection of miRNA mimics

MiR-133b mimic (dsRNA oligonucleotides) and negative control mimic (NC) (sense: 5′-UUC UCC GAA CGU GUC ACG UTT-3′, antisense: 5′-ACG UGA CAC GUU CGG AGA ATT-3′) were purchased from GenePharma (Shanghai, China). Transfection was carried out using Lipofectamine™ 2000 (Invitrogen) according to the manufacturer’s procedures. MiRNA mimics were used at a final concentration of 100 nM.

### Scratch assay

At 16 h post-transfection with miRNA mimics, cells (1 × 10^6^ cells/well) were seeded to 90% confluence in a 6-well plate for overnight culture. A scratch was made through the center of each well using a pipette tip, creating an open “wound” that was clear of cells. The dislodged cells were removed by three washes with culture media. Plates were then cultured with serum-reduced medium containing 1% FBS. Migration into the open area was documented at 72 h post-scratching. Each condition was tested in triplicate and each experiment was repeated at least three times.

### Cell migration and invasion assays

At 16 h post-transfection with miRNA mimics, 5 × 10^4^ cells in serum-free medium were introduced into the upper compartment of the BD BioCoat control inserts (Cat. # 354578, BD Discovery Labware, Bedford, MA, USA) fitted with membranes of 8 micron porosity separating the upper and lower compartments. The lower compartment was filled with normal culture medium supplemented with 10% FBS as the chemoattractant. Cells were incubated for 48 h for the migration assay and 72 h for the invasion assay. For the invasion assay, the inserts were previously coated with extracellular matrix gel (BD Biosciences, Bedford, MA, USA). At the end of the experiments, the cells on the upper surface of the membrane were removed, and the cells on the lower surface were fixed and stained with 0.2% crystal violet. Five visual fields of each insert were randomly chosen and counted under a light microscope. Each condition was assayed in triplicate and each experiment was repeated at least three times.

### Construction of the reporter gene system and luciferase activity assay

The 203 bp full length wild-type (WT) Gli1-3′UTR containing the putative miR-133b binding site or mutant Gli1-3′UTR (mut) was synthesized (Sangon, Shanghai, China). After digestion by SpeI and HindIII, the fragments of wild-type and mutant Gli1-3′UTR were cloned into the SpeI and HindIII sites of the pMIR-Report luciferase vector (Applied Biosystems) and named pMIR/Gli1 and pMIR/Gli1/mut, respectively. Sequencing was used to verify the constructs.

For the relative luciferase reporter assay, cells were seeded in a 24-well Plate 24 h prior to assay performance. In each well, 100 ng pMIR/Gli1 or pMIR/Gli1/mut, 1 ng pRL-TK (Promega, Madison, WI, USA) containing Renilla luciferase and 100 nM miRNA mimics were cotransfected using Lipofectamine™ 2000 reagent. Relative luciferase activity was calculated 48 h after cotransfection using the Dual-Glo Luciferase assay (Promega) according to the manufacturer’s procedure. Firefly luciferase activity was normalized to Renilla luciferase activity.

### Western blot analysis

Protein levels were quantified by standard western blot procedures with the following antibodies: Gli1 (1:1000, Cell Signaling Technology, Beverly, Massachusetts, USA), OPN (1:500, IBL, Japan), Zeb2 (1:1000, Prosci, Poway, CA, USA) and GAPDH (1:20000, Abcam, Cambridge, UK). Protein levels were normalized to total GAPDH levels.

### Retroviral transfection for stable cell lines

As previously described [8], retroviruses containing miR-133b or no insert (NC, negative control) were produced. After infections of MKN-28 cells, positive cells were selected and named RV-miR-133b and RV-miR-NC. MiR-133b expression was confirmed by qRT-PCR.

### *In vivo* metastasis peritoneal spreading assay

MKN-28, RV-miR-NC and RV-miR-133b cells were resuspended and injected intraperitoneally (2 × 10^6^ cells/mouse) into 4-week-old male BALB/C nude mice (Shanghai Laboratory Animal Center of China). Ten mice were included in each group. On the 60^th^ day after intraperitoneal injection, mice were euthanized by cervical dislocation, and peritoneal spreading of tumor lesions was assessed by necropsy. All experiments were performed in accordance with the official recommendations of the Chinese Animal Committee.

### Statistical analysis

All tests of significance were two tailed. Continuous variables were compared using the Student’s *t* test for normally distributed variables and Wilcoxon rank-sum test for non-normally distributed variables. The relationship between the miR-133b expression levels and clinicopathologic parameters was analyzed using tertiles and the Pearson Chi-square test. All values are presented as mean ± SD. All statistical analyses were performed using PASW Statistics 18.0 software (IBM, Chicago, IL, USA). p <0.05 was considered to indicate a statistically significant result.

## Results

### The expression of miR-133b is downregulated in GC

**Figure 1:**
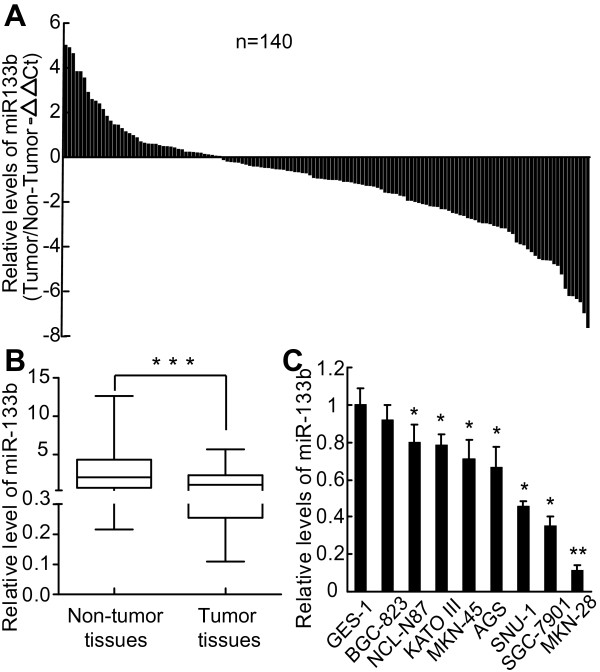
**MiR-133b is downregulated in GC tissues and cells. (A)** Relative expression of miR-133b in 140 GC patient tissues compared with adjacent non-tumor tissues. qRT-PCR results are shown as -△△CT values. **(B)** The boxes represent the distribution of miR-133b expression from the 25the to 75th percentiles of all patient samples. The whiskers represent distribution from the 10th to 90th percentiles, with the horizontal lines showing the median levels of miR-133b. ***, p <0.001. **(C)** Relative expression of miR-133b in eight GC cell lines and one immortalized normal gastric mucosal epithelial cell line (GES-1). qRT-PCR results are the mean of three independent experiments ± S.D. *, p <0.05, **, p <0.01.

Previous microarray results suggested that miR-133b was significantly downregulated in GC [6]. To confirm the microarray results, we examined paired tumor and adjacent non-tumor gastric tissues from 140 GC patients using qRT-PCR analysis. Expression of miR-133b was significantly downregulated in tumor tissues compared with matched non-tumor tissues in 70% (98/140) of the GC patients (p < 0.001) (Figure 1A, B). Cellular experiments found similar results, showing that expression of miR-133b was much lower in the seven tested GC cell lines than in the immortalized normal gastric mucosal epithelial cell line GES-1 (Figure 1C). Together these results provide strong evidence that miR-133b is markedly downregulated in GC.

To elucidate correlations between the expression level of miR-133b and clinicopathologic features in GC, the clinical and pathologic characteristics of the 140 GC cases were further analyzed (Table 1). The 140 cases were stratified into three groups based on relative miR-133b expression (tumor/non-tumor ratio) using tertiles: miR-133b low expression (tumor/non-tumor ratio < 0.24, n = 46), miR-133b moderate expression (tumor/non-tumor ratio 0.24–0.87, n = 47) and miR-133b high expression (tumor/non-tumor ratio > 0.87, n = 47). The miR-133b expression levels were negatively correlated to lymph node metastasis in these patients, with the miR-133b low-expression group exhibiting a significantly higher rate of lymph node metastasis rate compared with moderate- or high-expression groups (p = 0.03). And miR-133b expression levels also showed negative relationship with age. However, miR-133b expression levels did not show any relationship with gender, tumor differentiation, tumor location, tumor local invasion or TNM stage (Table 1).

**Table 1:** Relationship between expression levels of miR-133b and clinicopathologic parameters in 140 GC cases

**Clinicopathologic parameters**	**miR-133b expression**			**p-value**
	**Low (n = 46)**	**Middle (n = 47)**	**High (n =47)**	
Age (years)				
≤60	20	16	28	*0.04
>60	26	31	19	
Gender				
Male	39	30	31	0.05
Female	7	17	16	
Differentiation				
High, middle	14	17	9	0.18
Low	32	30	38	
Location				
Distal third	28	23	24	0.63
Middle third	13	14	16	
Proximal third	5	10	7	
Local invasion				
T1	5	6	1	0.16
T2	14	5	12	
T3	10	14	13	
T4	17	22	21	
Lymph node metastasis				
N0	7	12	13	*0.03
N1	7	7	11	
N2	14	19	19	
N3	18	9	4	
TNM stage				
I, II	22	20	22	0.86
III, IV	24	27	25	

A total of 140 cases were stratified into three groups based on relative miR-133b expression (tumor/non-tumor ratio) using tertiles: miR-133b low expression (tumor/non-tumor ratio < 0.24, n = 46), miR-133b moderate expression (tumor/non-tumor ratio 0.24–0.87, n = 47) and miR-133b high expression (tumor/non-tumor ratio > 0.87, n = 47). The relationship between the miR-133b expression levels and clinicopathologic parameters was analyzed using the Pearson Chi-square test. *, p <0.05.

### Overexpression of miR-133b inhibits metastasis of GC cell *in vitro*

The negative relationship between miR-133b and lymph node metastasis of GC aroused interest in the role of miR-133b in the metastasis of GC cells. The expression of miR-133b was the lowest in MKN-28 and SGC-7901 cells, so we selected these two cell lines as models to investigate metastasis *in vitro*. Synthetic miR-133b mimic and negative control mimic (NC) were transfected into MKN-28 or SGC-7901 cells respectively. The ectopic expression of miR-133b in cells was confirmed by qRT-PCR (Additional file 1: Figure S1).

**Figure 2:**
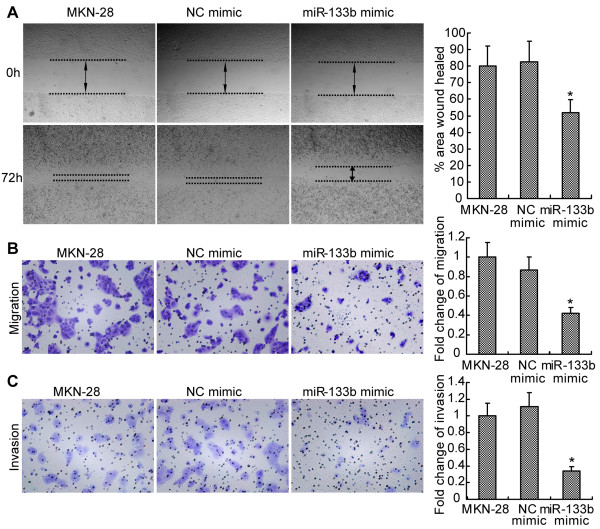
**MiR-133b inhibits metastasis of GC cells *in vitro*.** Representative images of scratch assays **(A)**, migration assays **(B)** and invasion assays **(C)** of MKN-28 cells, MKN-28 cells transfected with miR-133b mimic or negative control mimic (NC) (left panels). Relevant quantification of the results is shown in the bar graphs (right panels). The results are mean of three independent experiments ± S.D. *, p <0.05.

As shown in Figure 2A, cell migratory ability was significantly inhibited in MKN-28 cells transfected with miR-133b mimic. The ability of MKN-28 cells to migrate through an insert membrane was also significantly inhibited by miR-133b (Figure 2B), and cell invasion through the extracellular matrix gel was also reduced by miR-133b (Figure 2C). Similar results were observed in SGC-7901 cells (Additional file 2: Figure S2). Results in both cell lines showed the repression of metastasis *in vitro* by overexpression of miR-133b.

### Overexpression of miR-133b inhibits peritoneal spreading of GC cells *in vivo*

**Figure 3:**
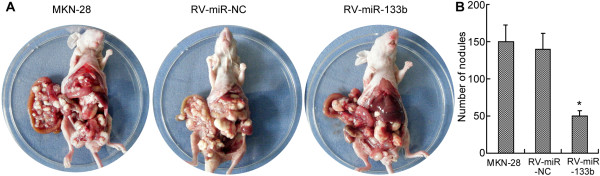
**MiR-133b inhibits peritoneal spreading in nude mice. (A)** Representative images of nude mice injected with indicated cells. **(B)** Quantification of the peritoneal nodules is shown in the bar graph. The results are mean of 6–10 mice ± SD. *, p <0.05.

Given that miR-133b inhibited metastasis of GC cells *in vitro*, we further tested whether miR-133b could affect tumor metastasis *in vivo*. MKN-28 cells, RV-miR-133b (MKN-28 cells with retrovirus-mediated miR-133b stable expression) or RV-miR-NC (MKN-28 cells with the empty vector) were obtained as described in the Methods section. After miR-133b expression in the stable cell lines was confirmed by qRT-PCR (Additional file 3: Figure S3), cells were intraperitoneally injected into 4-week-old male nude mice. The mice were euthanized two months after the injection, and the tumor lesions in the peritoneal cavity were counted. The number of peritoneal nodules was significantly less in mice injected with RV-miR-133b cells than in the MKN-28 group or RV-miR-NC group (Figure 3). Thus, these results indicate that miR-133b could suppress metastasis of GC cells *in vivo*.

### Gli1 is a target gene of miR-133b

To identify how miR-133b functions in GC cells, computational prediction of miR-133b targets was performed. We used the online search tools miRNAMap 2.0 [25], which employs miRanda [26], TargetScan [27] and RNAhybrid [28], as computational tools. Among the hundreds of candidate genes predicted by both of miRanda and RNAhybrid, the transcription factor Gli1 was of particular interest. Gli1 has been reported as highly expressed in GC and positively correlated with lymph node metastasis [29,30]. Furthermore, a previous study showed that Gli1 siRNA reduced GC cell migration and invasion, possibly through SIP1 [31] or PI3K/Akt pathway [30].

**Figure 4:**
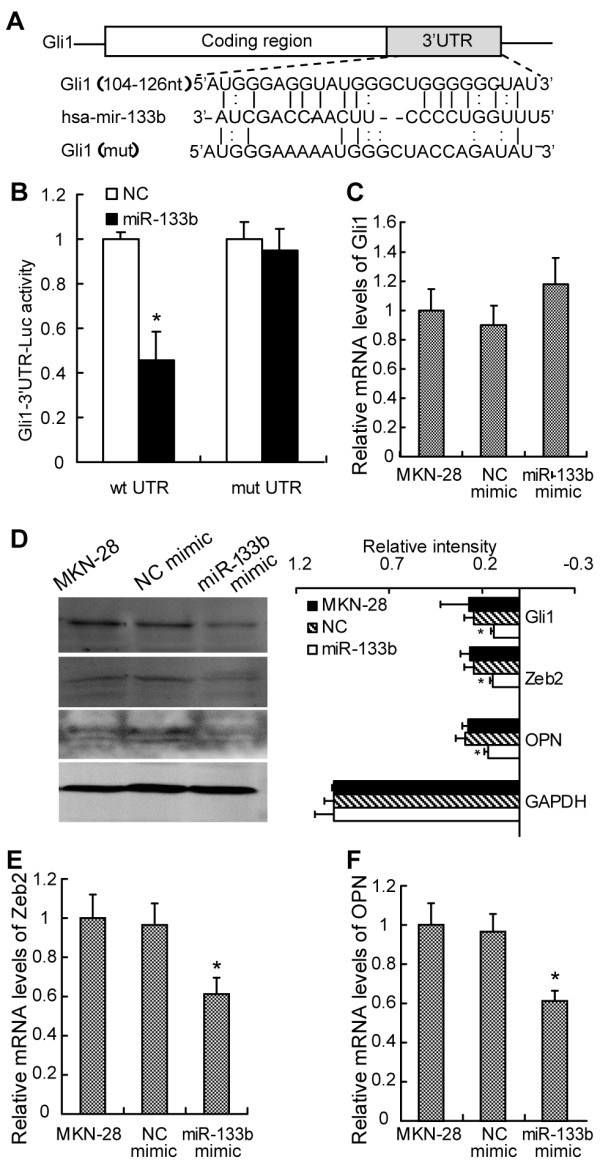
**MiR-133b direct targets Gli1 in GC cells. (A)** Sequence of the Gli1 3′UTR showing the miR-133b binding seed region and mutation of the Gli1 3′UTR seed region to create Gli1-mut. **(B)** Luciferase activities of the reporter containing wild-type Gli1 3′UTR or mutant Gli1 3′UTR are shown in the bar graph. **(C)** Relative mRNA levels of Gli1 in indicated cells analyzed by qRT-PCR is shown in the bar graph. **(D)** Representative western blot images of indicated protein in MKN-28 cells (left panels), with relevant quantification (right panel). Relative mRNA levels of Zeb2 **(E)** and OPN **(F)** in indicated cells analyzed by qRT-PCR are shown in the bar graph. The results are the mean of three independent experiments ± S.D. *, p <0.05.

Luciferase reporter assays were performed to verify a possible direct interaction between miR-133b and the 3′UTR of Gli1. Luciferase reporters were constructed containing either wild-type full-length Gli1 3′UTR (pMIR/Gli1) or a mutated Gli1 3′UTR (pMIR/Gli1/mut, in which the sequence of the putative miR-133b binding site was mutated) (Figure 4A). The pMIR/Gli1 and pMIR/Gli1/mut luciferase reporter constructs were transfected into MKN-28 cells along with miR-133b or negative control mimic. The relative luciferase activity of the pMIR/Gli1 reporter was markedly suppressed by 45.6% (p < 0.01) compared with that of pMIR/Gli1/mut in a miR-133b-dependent manner (Figure 4B). This result strongly indicates that the 3′UTR of Gli1 harbors a direct binding sites for miR-133b.

To examine whether miR-133b affects Gli1 at mRNA level or protein level, Gli1 expression was examined by qRT-PCR and western blot. As shown in Figure 4C, miR-133b mimic had no effect on Gli1 mRNA level. However, Gli1 protein level was decreased in miR-133b mimic-transfected MKN-28 cells as shown in Figure 4C. These results strongly suggest that miR-133b negatively regulates Gli1 expression through translation repression rather than mRNA degradation.

Among the genes reported to promote the metastasis of GC [32-34], Zeb2 and OPN are direct transcriptional targets of Gli1 [35,36]. As shown in Figure 4E and F, the mRNA levels of Zeb2 and OPN were both markedly reduced in MKN-28 cells transfected with miR-133b mimic. Protein levels of Zeb2 and OPN also were markedly reduced in MKN-28 cells transfected with miR-133b mimic (Figure 4D).

## Discussion

Several findings have linked miRNAs to GC. MiR-133b, located in chromosome 6, was predicted based on comparative analysis of human, mouse and Fugu [37], and experimentally verified by sequencing in 2007 [38]. Although miR-133b was originally suggested to be solely expressed in skeletal muscle [14], it was suggested to act as a tumor-suppressor in many types of cancers recently [15-22]. In this study, we found that miR-133b was frequently decreased in the tumor tissues of GC patients, as well as in cultured GC cell lines, which is consistent with another group’s finding from miRNA microarray data in three GC patient tissues [13]. Importantly, miR-133b levels were negatively correlated with lymph node metastasis of gastric cancer in the 140 cases, which is consist with Wu’s finding in 15 lymph node negative GC tissues compare with 15 lymph node positive GC tissues [39].

Given that miR-133b was downregulated in GC tissues and negatively correlated with lymph node metastasis of GC, we speculated that overexpression of miR-133b might suppress metastasis of GC cells. Restoration of miR-133b in MKN-28 and SGC-7901 cells significantly inhibits metastasis both *in vitro* and *in vivo*. These results strongly suggested an inhibitory role of miR-133b in metastasis of GC, which is a novel finding. These results also strongly demonstrated that the decreased miR-133b expression in GC should be a factor contributing to the development of GC rather than being a consequence of GC. Therefore, the significant inhibition of peritoneal spreading in nude mice implies that therapeutic strategies of introducing miR-133b into cancer cells might be useful for slowing the process of tumorigenesis.

Identifying miRNA targets that are essential for cancer development and metastasis may help elucidate their mechanisms of action and the patheways that miRNAs modulate [40]. Using bioinformatic algorithms, we identified Gli1 as a possible direct target gene for miR-133b. Gli1 was initially found as an amplified gene in a malignant glioma [41]. It is a strong positive activator of downstream target genes and is a transcriptional target of Hedgehog signaling [42]. Gli1 can also be upregulated by RAS/PKC [43], TGFβ [44] and PI3K [45], and downregulated by PKA [45] and p53 [46]. Gli1 expression in epithelial cells can induce cell transformation characterized by anchorage-independent proliferation [47]. It has also been reported as a metastatic oncogene [36,48,49]. Increasing number of studies show that expression of Gli1 is upregulated in GC [50-52]. We validated this suggestion with luciferase reporter assays. MiR-133b directly suppressed expression of Gli1 in MKN-28 cells, which occurred through translation repression rather than mRNA degradation. Furthermore, the expression levels of Gli1 downstream target genes Zeb2 and OPN [35,36] were decreased. Zeb2 and OPN were reported to promote the metastasis of GC [32-34]. This suggests that GC cell metastasis inhibition induced by miR-133b might be partially related to its suppression of Zeb2 and OPN expression, which occurs via direct interaction with the Gli1 3′UTR. Taken as a whole, these data indicate that miR-133b suppresses GC metastasis at least partially through direct interaction with the Gli1 3′UTR. In addition to our finding, Wen et al. reported that miR-133b could inhibit GC cell proliferation and colony formation *in vitro* by direct targeting FGFR1 [53]. It is likely that as a novel tumor suppressor, miR-133b has multiple targets and functions in GC tumor cells. Further studies are needed to fully understand the role of miR-133b in tumor metastasis.

## Conclusions

In summary, miR-133b is frequently decreased in human gastric cancer. Restoration of miR-133b inhibits GC metastasis, at least partly by directly suppressing the expression of Gli1. Such roles for miR-133b in GC suggest its potential as a therapeutic microRNA for GC treatment, which is worth further investigation.

## Abbreviations

miRNA: MicroRNA; GC: Gastric cancer; 3′UTR: 3′-untranslated regions; qRT-PCR: Quantitative real-time PCR.

## Competing interests

The authors declare that they have no competing interests.

## Authors’ contributions

ZZ and BL conceived the study design, participated in its design and in the acquisition of data. YZ carried out the experiments, participated in the acquisition of data, analysis and interpretation, drafted the manuscript. JH, LZ, YQ has been involved in analyzing the data and drafting the manuscript. JL, BY, MY, YY helped to draft and revise the manuscript. All authors read and approved the final manuscript.

## Pre-publication history

The pre-publication history for this paper can be accessed here:

http://www.biomedcentral.com/1471-2407/14/34/prepub

## Supplementary Material

Additional file 1: Figure S1 MiR-133b mimic significantly enhanced miR-133b level in MKN-28 and SGC-7901 cells. Relative levels of miR-133b in MKN-28 (A) and SGC-7901 cells (B) were analyzed by qRT-PCR and shown in the bar graph. The results are the mean of three independent experiments ± S.D. ***, p < 0.001. Click here for file

Additional file 2: Figure S2 MiR-133b inhibits metastasis of SGC-7901 cells *in vitro*. Representative images of scratch assays (A), migration assays (B) and invasion assays (C) of SGC-7901 cells,SGC-7901 cells transfected with the miR-133b mimic or negative control mimic (NC) (left panels). Relevant quantification is shown in bar graphs (right panels). The results are the means of three independent experiments ± S.D. *, p < 0.05. Click here for file

Additional file 3: Figure S3 Expression of miR-133b in stable cell lines. Relative levels of miR-133b in MKN-28, RV-miR-NC and RV-miR-133b cells were analyzed by qRT-PCR and shown in the bar graph. The results are the means of three independent experiments ± S.D. ***, p < 0.001. Click here for file
