# Supplementary figures and images for: MiR-133b is frequently decreased in gastric cancer and its overexpression reduces the metastatic potential of gastric cancer cells
Source: BMC Cancer. 2014 Jan 21;14:34. doi: 10.1186/1471-2407-14-34 (PMC3925791; doi:10.1186/1471-2407-14-34)

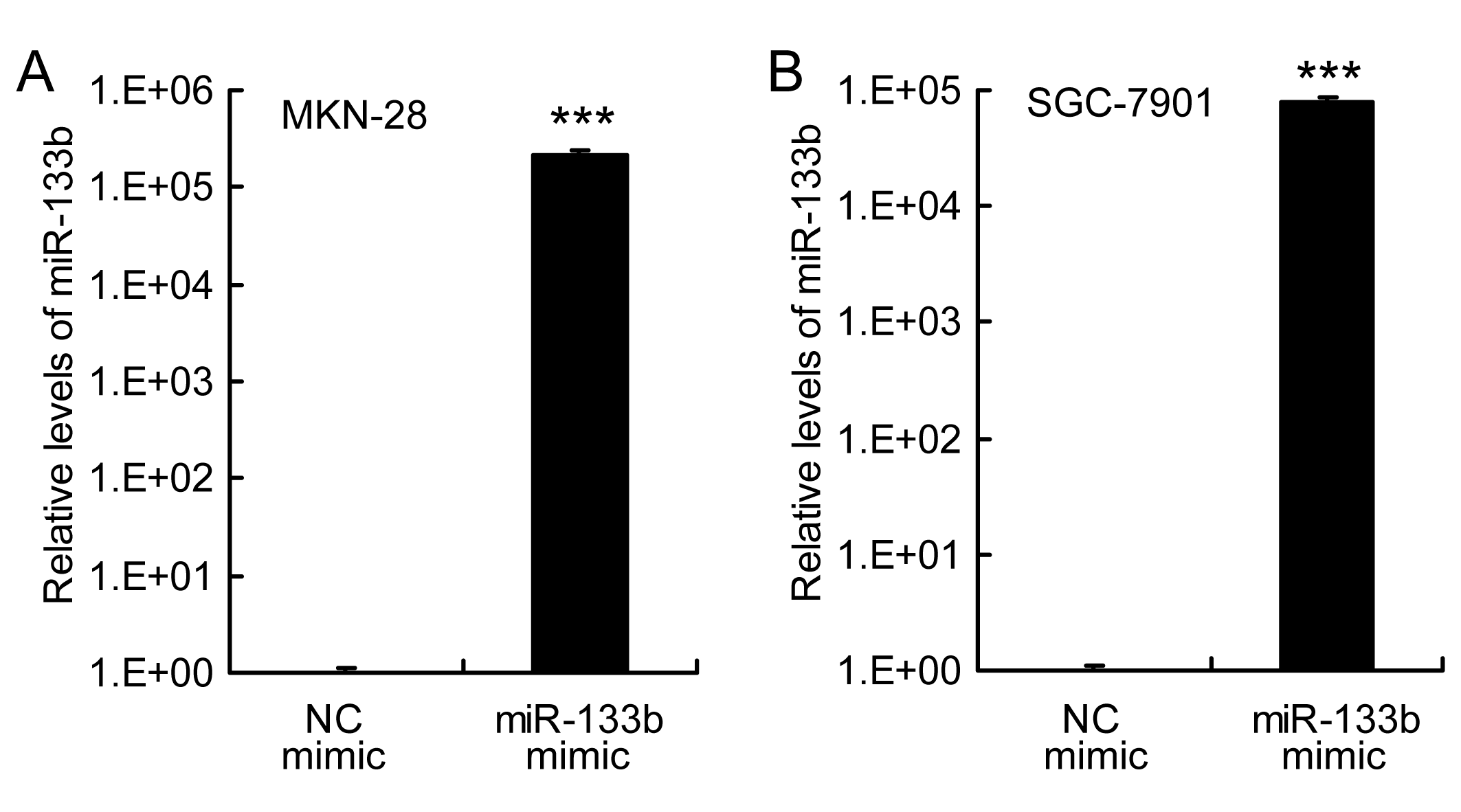

Supplement: Additional file 1: Figure S1 — MiR-133b mimic significantly enhanced miR-133b level in MKN-28 and SGC-7901 cells. Relative levels of miR-133b in MKN-28 (A) and SGC-7901 cells (B) were analyzed by qRT-PCR and shown in the bar graph. The results are the mean of three independent experiments ± S.D. ***, p < 0.001. [file 1471-2407-14-34-S1.tiff]

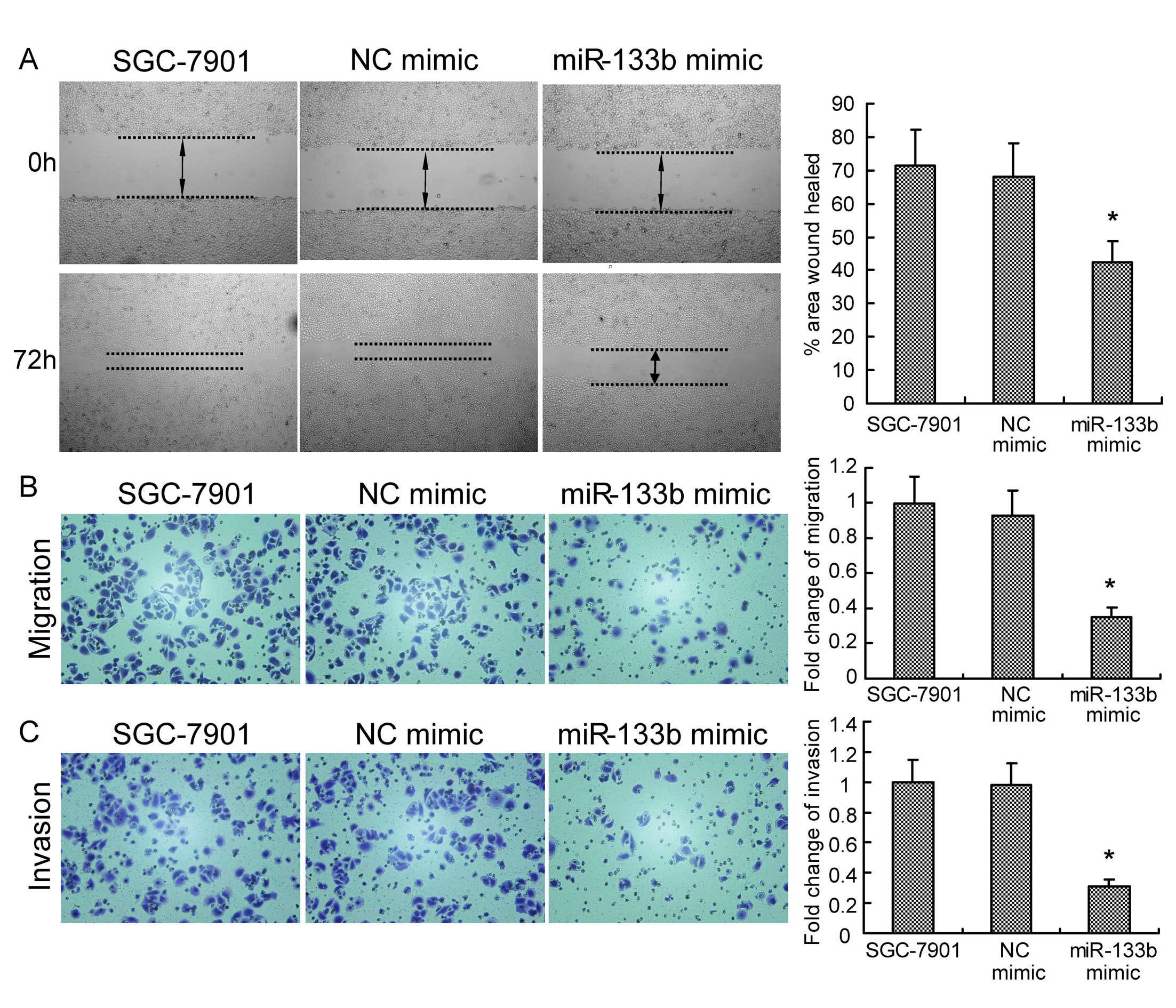

Supplement: Additional file 2: Figure S2 — MiR-133b inhibits metastasis of SGC-7901 cells in vitro. Representative images of scratch assays (A), migration assays (B) and invasion assays (C) of SGC-7901 cells,SGC-7901 cells transfected with the miR-133b mimic or negative control mimic (NC) (left panels). Relevant quantification is shown in bar graphs (right panels). The results are the means of three independent experiments ± S.D. *, p < 0.05. [file 1471-2407-14-34-S2.jpg]

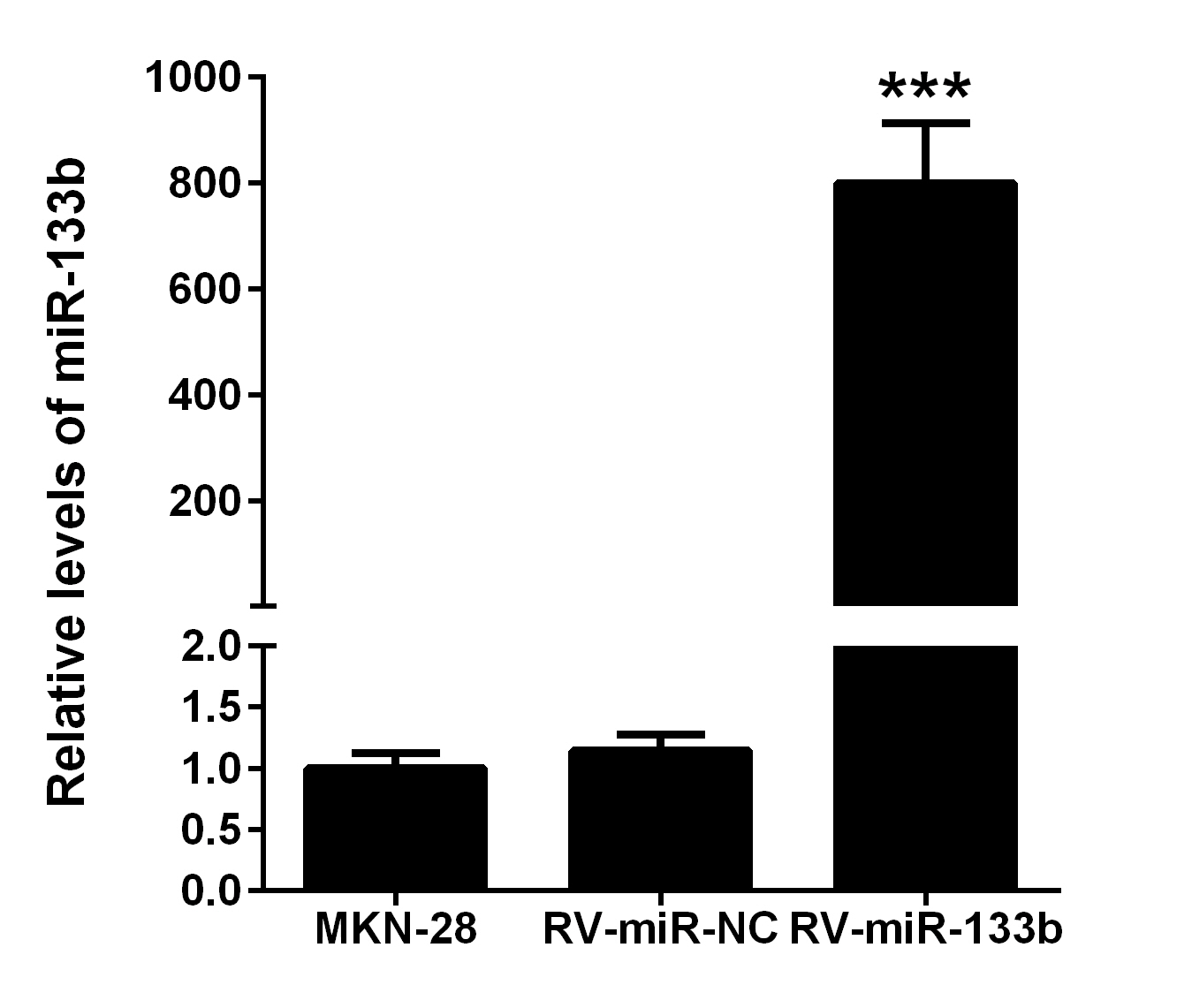

Supplement: Additional file 3: Figure S3 — Expression of miR-133b in stable cell lines. Relative levels of miR-133b in MKN-28, RV-miR-NC and RV-miR-133b cells were analyzed by qRT-PCR and shown in the bar graph. The results are the means of three independent experiments ± S.D. ***, p < 0.001. [file 1471-2407-14-34-S3.jpeg]
